# Valuing the “Burden” and Impact of Rare Diseases: A Scoping Review

**DOI:** 10.3389/fphar.2022.914338

**Published:** 2022-06-08

**Authors:** Julien Delaye, Pasquale Cacciatore, Anna Kole

**Affiliations:** ^1^ European Organisation for Rare Diseases (EURORDIS), Paris, France; ^2^ GSK Vaccines Srl, Siena, Italy

**Keywords:** Quality of Life, Cost of illness, Rare diseases, Impact Assesment, burden of disease, Scoping review, Europe

## Abstract

**Introduction:** Rare diseases (RDs) are a severe, chronic, degenerative and often life-threatening group of conditions affecting more than 30 million people in Europe. Their impact is often underreported and ranges from psychological and physical symptoms seriously compromising quality of life. There is then a need to consolidate knowledge on the economic, social, and quality of life impacts of rare diseases.

**Methods:** This scoping review is the result of 9 qualitative interviews with experts and a literature search on Cost-of-Illness (COI) studies and quality of life (QoL) studies following the PRISMA methodology. Grey literature was also included to complement findings. Results. 63 COI studies were retrieved, covering 42 diseases and a vast majority of them using a prevalence-based approach (94%). All studies included medical costs, while 60% included non-medical costs, 68% productivity losses and 43% informal care costs. 56 studies on QoL were retrieved, mostly from Europe, with 30 different measurement tools. Grey literature included surveys from the pharmaceutical industry and patient organisations.

**Discussion:** The majority of studies evaluating the impact of RDs on the individual and society use the COI approach, mostly from a societal perspective. Studies often vary in scope, making them difficult to consolidate or compare results. While medical costs and productivity losses are consistently included, QoL aspects are rarely considered in COI and are usually measured through generic tools.

**Conclusion:** A comprehensive study on impact of rare disease across countries in Europe is lacking. Existing studies are heterogeneous in their scope and methodology and often lack a holistic picture of the impact of rare. Consensus on standards and methodology across countries and diseases is then needed. Studies that consider a holistic approach are often conducted by pharmaceutical companies and patient organisations exploring a specific disease area but are not necessarily visible in the literature and could benefit from the sharing of standards and best practices.

## Introduction

Rare diseases are a group of an estimated 6,000 to 9,000 known severe, chronic, degenerative, and often life-threatening conditions defined as diseases affecting no more than 1 in 2,000 people in Europe (Sequeira et al., 2021). Although each of these diseases affects a small number of people, it is estimated that approximately 30 million individuals are living with a rare disease in Europe, the vast majority of them being of genetic origin and typically associated with reduced life expectancy and loss of quality of life, with symptoms ranging from physical to psychological impacts seriously compromising day-to-day activities, autonomy and well-being (Uhlenbusch et al., 2019a). Over the past decades, rare diseases have progressively been acknowledged as an important public health issue, greatly impacting the lives of people living with such conditions, their family and caregivers, healthcare systems and society. However, while research is crucial to understand these conditions and to address their associated challenges, knowledge on the impact they represent is still largely limited in the literature. The majority of research tends to focus on their economic impact, while the political and public discourses are mostly driven by the often very high prices of their treatments and management (Vogler et al., 2017). For some very costly treatments, such as gene therapy, health technology assessments (HTAs) must balance price with its potential benefits for people living with a rare disease (Kolata, 2014). Even for less transformative therapies, evaluating the impact of rare diseases beyond economic perspective must be considered to accurately depict and take account of challenges faced by people living with rare diseases and to highlight care opportunities to address them. With the emergence of new technologies and constant innovation in care, it is likely that discussions around the financing, assessment, access to and reimbursement of such treatments and care options will become increasingly salient in public and political spheres and will require robust evidence-based analyses for effective and fair decision-making processes. Above all, the multifaceted and heterogeneous character of rare diseases, coupled with the multitude of impacts they have on patients, society and healthcare systems, leads to different ways to evaluate their impacts. Depending on the perspectives and interests of those conducting the impact assessment of rare diseases, different conclusions may be drawn and findings in the literature may therefore greatly vary from one discipline to another. To be truly representative, studies investigating the impact of rare diseases should thus take account of all these perspectives to provide interested parties with a comprehensive analysis of rare diseases and their implications in the life of people living with these conditions.

### Defining Impact

The burden of disease is used to evaluate the impact of diseases and, depending on the researcher’s field, typically considers clinical, economic, and/or political indicators and is usually expressed in terms of the costs a disease and/or disability exert upon the individual, healthcare system or society. As defined by the World Health Organisation (WHO) (World Health Organisation, 2021) and the Centres for Disease Control and Prevention (CDC) (Centers for Disease Control and Prevention, 2021), the burden of disease describes death and loss of health due to diseases, injuries, and risks factors. However, the definition of “burden” may differ from one discipline to another and include different aspects relevant to the discipline in question, including methodologies used to evaluate it such as Disability Adjusted Life Years (DALYs), Cost-of-Illness (COI) analysis and Health-Related Quality of Life (HRQoL) measurements. There have been attempts to assess the burden of rare diseases over time, most of them being disease and country specific. For example, the BURQoL-RD (Burqol-Rd Project, 2013) project addressed the socioeconomic impact of 10 rare diseases in 8 European countries. More recently, the EveryLife Foundation (EveryLife Foundation, 2021) in the US investigated the economic burden of 379 rare diseases in its National Economic Burden of Rare Disease Study, the first report to include such a large number of conditions so far. Similar initiatives are currently missing in Europe, where the complexity and heterogeneity of national contexts further complicate the assessment of the burden of rare diseases beyond borders and across pathologies. In a recent scoping review on this topic (García-Pérez et al., 2021), have shown that COI analyses are the most prevalent approach when assessing the burden of rare diseases. Defined as a type of economic study that identifies and measures all the costs of a particular disease, these analyses usually include direct medical costs (e.g. in/outpatient care, physician visits), indirect/productivity (e.g. absenteeism, forced/early retirement.) and non-medical costs (e.g. daily care, education-related costs). However, the challenges inherent to the definition of rare diseases, such as the limited amount of available primary and aggregated data, is known to be an issue in these analyses (García-Pérez et al., 2021). Angelis et al. (Angelis et al., 2015) also postulate that comparison of results of COI studies in rare diseases is often hampered due to a diversity of designs and methodologies, while aspects such as quality of life seem to be falling outside the scope of COI analyses and thus not consistently included in burden of disease studies.

The goal of this paper is therefore to review how the burden of disease has been measured to date, including differences in methodologies used to do so, to identify current knowledge gaps, and to propose recommendations on how the impact of rare disease should be measured in future research to be better adapted to their nature. A scoping review of the peer reviewed, policy and grey literature was thus conducted and offers research and policy directions to capture the true impact of rare diseases, encompassing all costs and features impacting people living with a rare disease, healthcare systems and society. Despite the use of the word “burden” in the literature, the authors of this paper will use the term “impact” to refer to the burden of disease, as burden tends to be disliked and negatively perceived by the rare disease community.

## Methods

### Eligibility Criteria

Rare diseases included in the scope of this paper were selected regardless of their severity or amount of data available on these diseases. Sources were excluded from the selection when a language other than English or French was used. The scope of the literature search was however not geographically limited and included sources published between 2000 and 2021. Authors selected studies and articles that focused on rare diseases and addressed the topic of the impact of rare diseases, including both COI studies on the basis of a previous review (García-Pérez et al., 2021) and other studies encompassing aspects falling outside the scope of COI (quality of life, well-being, mental health). Characteristics of COI studies used for the purpose of this review were directly retrieved from the scoping review conducted by García-Pérez et al. (García-Pérez et al., 2021), from which authors drew their own conclusions. As for sources on quality of life (QoL) and health-related quality of life HRQoL, only studies measuring QoL in rare disease populations were included, thus excluding scoping, narrative, and systematic reviews, as well as methodological articles. The goal of this paper being to assess the available literature on the impact of rare diseases altogether, the selection of sources included disease-specific and non-disease-specific sources. Rare diseases included in the scope of this paper were selected regardless of their severity or amount of data available on these diseases. The criteria for inclusion of conditions in the scope of this review was the European prevalence threshold of less than 1 person in 2,000 (Sequeira et al., 2021). Sources were excluded from the selection when a language other than English or French was used.

### Information Sources

As mentioned earlier, the impact of disease can be defined in different ways depending on the discipline and perspective it is studied from. For this reason, this scoping review was therefore based on a two-tied approach, which allowed for the inclusion of the different relevant perspectives: a qualitative phase and a literature search. Qualitative structured interviews were first conducted with experts in the field of rare diseases who are currently or have been conducting research on the impact of disease to guide the authors in 1. defining the appropriate scope and methods for conducting the scoping review, 2. refining their search and selection of appropriate keywords and 3. contributing to the identification and collection of relevant literature. Especially regarding intangible costs of rare diseases, these experts were consulted to identify the most common and appropriate measurement tools, which were later compared with findings in the literature. The literature search was conducted between April 2021 and October 2021. Peer-reviewed articles were identified and retrieved from Google Scholar and PubMed using keywords (“rare diseases”, “cost-of-illness”, “socioeconomic”, “burden of diseases”, “quality of life”). Authors also included grey literature in the scope of this review. Grey literature was identified and retrieved thanks to the experts and network consulted during the qualitative phase of this review and *via* Google’s search engine.

### Selection of Sources of Evidence, Data Charting Process and Synthesis of Results

One author (Julien Delaye) conducted the initial literature search by screening sources’ titles and, when titles were in line with the scope of this review, abstracts. A second author (Anna Kole) reviewed the selection of articles and studies, added further sources (both peer-reviewed and grey literature), commented on sources’ relevance until consensus was reached on the final selection for the production of the article. The third co-author (Pasquale Cacciatore) reviewed the article at a later stage and suggested amendments. Characteristics of the retrieved sources on peer-reviewed COI are geographic area, disease, prevalence or incidence-based estimation, perspective, and costs included as per García-Pérez et al. (García-Pérez et al., 2021). Characteristics of the retrieved sources on quality of life are diseases investigated, geographical area and measurement tools. Characteristics of retrieved grey literature sources are diseases investigated when specific to one rare disease, measurement tools and initiators of the project/paper/survey. In line with the scope of this review, authors did not analyse economic specificities and findings but rather aimed at presenting an overview of the studies’ and sources’ characteristics. Quality, biases, and limitations of the retrieved sources were not discussed.

## Results

### Selection of Sources of Evidence

**FIGURE 1:**
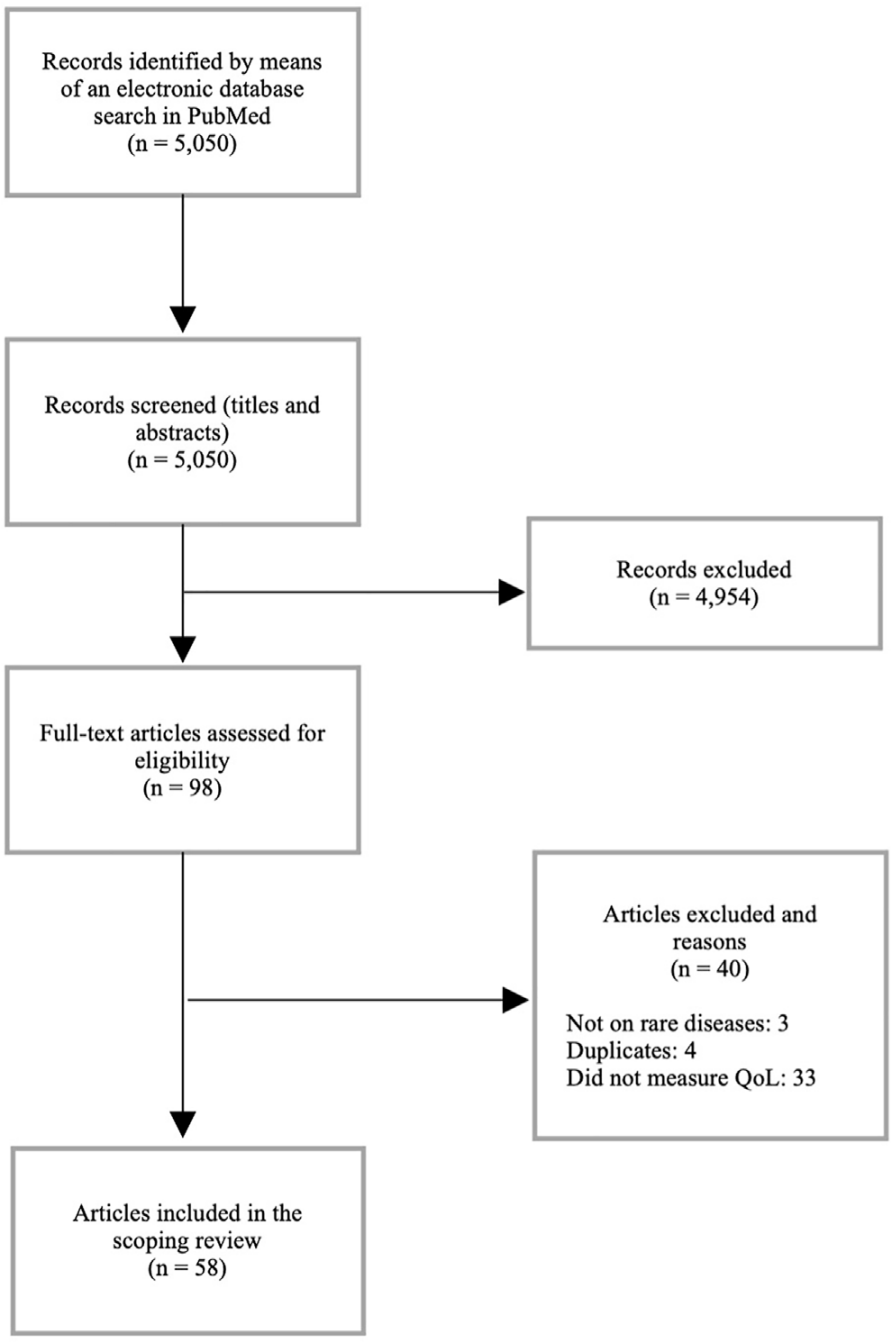
Flow diagram of study selection.

Peer-reviewed sources on the topic of COI were extracted from the scoping review conducted by García-Pérez et al. (García-Pérez et al., 2021), comprising 63 COI studies, and additional literature on epidemiological, clinical and economic aspects of acromegaly in Bulgaria (Kamusheva et al., 2020) and on Cri du Chat Syndrome in Italy (Kodra et al., 2020). The literature search on (health-related) QoL on PubMed identified 5.050 peer-reviewed references. The initial screening of titles and abstracts allowed authors of this paper to retrieve 98 sources for eligibility assessment, of which 40 sources were eventually excluded from the scope of this review due to several reasons, as shown in Figure 1. As for grey literature, 7 sources were retrieved to complement findings.

### Characteristics of Sources of Evidence

The 65 studies (see Table 1) included in this review covered 43 rare diseases, of which 10 rare diseases were investigated in more than one study: amyotrophic lateral sclerosis (ALS), haemophilia, Duchenne muscular dystrophy (DMD), cystic fibrosis (CF), chronic inflammatory demyelinating polyradiculoneuropathy, idiopathic pulmonary fibrosis, juvenile idiopathic arthritis (JIA), Prader-Willi syndrome, systemic sclerosis, and tuberous sclerosis complex. COI studies were conducted in 25 countries, with a predominance of European countries (17 countries) covered by retrieved papers, which García-Pérez et al. (García-Pérez et al., 2021) partly attribute to the BURQoL-RD project. The influence of the BURQoL-RD project was also reflected in the number of studies available for specific diseases and disease groups (e.g. Haemophilia, DMD, CF). The vast majority of studies (94%) used prevalence-based estimations, while the remaining sources were incidence-based estimations on a patient’s lifetime horizon. The totality of studies reviewed included medical costs (inclusion of medical costs being an inclusion criteria of the review). 60% included non-medical costs, 69% productivity losses, and 43% included informal care. However, depending on the authors’ definition of informal care, this type of cost was classified as productivity losses in several studies, mostly with regards to parents and caregivers. A majority of studies also included more than one type of cost, as reflected in the abovementioned findings. In terms of perspectives, 63% of sources used a societal standpoint, while 27, 11 and 5% used healthcare systems, patients/families and hospitals perspectives respectively. Data were collected through questionnaires to patients and caregivers for 66% and/or extracted from medical charts or other types of databases (e.g. insurance claims, registries) (48%), some studies having combined both approaches, and another 6% having used a prospective data collection approach. Grey literature investigating the topic of the burden of disease through a COI methodology includes the National Economic Burden of Rare Disease Study (EveryLife Foundation, 2021) and an article investigating the impact of Inherited Retinal Diseases in the Republic of Ireland (ROI) and the United Kingdom (UK) from a cost-of-illness perspective (Galvin et al., 2020). The former includes medical, indirect and non-medical costs through a prevalence-based approach in the US, while the latter investigated data collected from COI reports in the ROI and the UK in a patient-centred standpoint. Similarly, Andreu et al. (Andreu et al., 2022) conducted the study “The Burden of Rare Diseases: an economic Evaluation” where they investigated the direct, indirect, and mortality cost for a sample of 24 rare diseases in the US. Lastly, a survey commissioned by Shire Human Genetic Therapies (Hendriksz, 2013) also investigated the costs of living with a rare disease alongside intangible costs through the use of a survey to patients and caregivers, physicians, tough leaders (policymakers, researchers, advocates) and payers.

**TABLE 1:** Characteristics of the 65 reviewed COi studies.

Characteristics	Number of studies
Prevalence/incidence-based estimation	
Prevalence	61 (94%)
Incidence	4 (6%)
Types of costs	
Medical costs	65 (100%)
Non-medical costs	39 (60%)
Lost productivity costs	45 (69%)
Informal care costs	28 (43%)
Perspectives	
Society	41 (63%)
Thirdpayer/healthcare system/government	18 (27%)
Patients and families	7 (11%)
Hospitals	3 (5%)
Sources of data	
Questionnaires	43 (66%)
Registries or databases	31 (48%)
Other	4 (6%)

The 58 retrieved peer-reviewed articles (Pasculli et al., 2004; López-Bastida et al., 2008; López-Bastida et al., 2009; Winter et al., 2010; Mirabel et al., 2011; Chhina et al., 2012; Calvert et al., 2013; Johansen et al., 2013; Kamusheva et al., 2013; Raluy-Callado et al., 2013; Johnson et al., 2014; Kodra et al., 2014; López Bastida et al., 2014; Poon et al., 2014; Bosch et al., 2015; Chevreul et al., 2015; Guffon et al., 2015; Verkaeren et al., 2015; Cavazza et al., 2016a; Cavazza et al., 2016b; Chevreul et al., 2016a; Chevreul et al., 2016b; Angelis et al., 2016; Iskrov et al., 2016; Landfeldt et al., 2016; López-Bastida et al., 2016a; López-Bastida et al., 2016b; Kuhlmann et al., 2016; Péntek et al., 2016; Zhang et al., 2016; Bogart and Irvin, 2017; Haghpanah et al., 2017; López-Bastida et al., 2017; van Walsem et al., 2017; Liu et al., 2018; Underbjerg et al., 2018; Wiemann et al., 2018; Bozovic et al., 2019; Hanisch et al., 2019a; Hanisch et al., 2019b; Lauby et al., 2019; Matos et al., 2019; Witt et al., 2019; Andersen et al., 2020; Applebaum et al., 2020; Berrocoso et al., 2020; Boettcher et al., 2020; Gao et al., 2020; Hanisch et al., 2020a; Hanisch et al., 2020b; Jansen et al., 2020; Kodra et al., 2020; Weidema et al., 2020; Chen et al., 2021; Giusti et al., 2021; Lee et al., 2021; Xu et al., 2021) (see Table 2) addressing the topic of QoL in rare disease covered 69 conditions, a few of them having included more than one pathology in their scope. The vast majority of diseases (51) were however covered by studies investigating only one disease. Most of the retrieved studies were conducted in Europe: Germany (23), Spain (18), France (16), Italy (15), the UK (13), Sweden (11), Bulgaria (10), Hungary (7), Norway (3), the Netherlands (1), Serbia (1) and Belgium (1). Similar studies were also found in the US (8), China (4), Brazil (2), Canada (2) and (South) Iran (1). Some of these studies included both European and non-European countries and two studies were not geographically limited (recruitment of participants achieved through Facebook and registries). A significant number of sources considered more than one country in their scope (e.g. BURQol-RD). The higher number of studies conducted in Germany, Spain, France, Italy, the UK, Sweden, Bulgaria, and Hungary is likely to be the result of the BURQoL-RD project that included these countries in their scope of action. 30 different measurement tools were used in the retrieved studies to measure (health-related) quality of life in rare diseases. Among these 30 tools, some were subdivided into more specific categories to better fit the purpose of the research or to use an updated version of the instrument (e.g. EQ-5D, EQ-5D-3L, EQ-5D-5L, SF-36, SF-12, S8, SF-6D). The most salient instruments were the combination of EQ-5D, Barthel Index and Zarit Scale (14), followed by Short-Form (including SF-36, SF-12, S8, SF-6D) (14), EQ-5D (including EQ-5D-3L and EQ-5D-5L) (9), PedsQL (including Peds.4.0) (8), and OHIP-14 (5). While the majority of tools were generic, 4 disease-specific measurement tools were also identified in the retrieved studies (Haemo-QoL questionnaire, AcroQoL, PKU-QoL and HS-Focus), the four of them having been used in one study each. The BURQoL-RD project again influenced these results, as the combination of EQ-5D, Barthel Index and Zarit Scale was used in all studies included in the project. Similar observations can be drawn from the use of PedsQL and OHIP-14 as these measurement tools were designated for studies investigating children and oral health respectively and specifically. As for grey literature, the IMPACT Survey (Osteogenesis Imperfecta Federation Europe, 2022) developed by Osteogenesis Imperfecta Federation Europe in collaboration with Mereo BioPharma focused on impacts of Osteogenesis Imperfecta on patients and their parents using a disease-specific and custom-made questionnaire. Another international and cross-sectional survey conducted by Ipsen (Ipsen, 2021) addressed impacts of Fibrodysplasia Ossificans Progressiva (FOP) on patients and their families through the use of a combination of disease-specific questionnaire (FOP Physical Function Questionnaire) and generic measurement tools (EQ-5D, PROMIS, Zarit Scale, Patient-Reported Mobility Assessment). The EURORDIS’ Juggling Care Survey (Eurordis, 2017) also provides insight into the impacts of rare diseases on patients and related-needs, impact on daily life (including for carers), coordination of care, access to services, work-life balance, and impacts on well-being and mental health through an online survey.

**TABLE 2:** QoL measurement tools used in reviewed QoL studies.

Measurement tool	Number
Short Form (incl. 36, 12, 8, 6D)	14
EQ-50 + Bathe] Index + Zarit Scale	14
EQ-5D (incl. -3L, -5L)	9
PedsQL (incl. Peds4.0)	8
OHIP-14	5
WHOQOL (BREF)	3
HAEMO-QoL Questionnaire	2
AcroQoL	2
Zarit Scale	2
Health Utilities Index Questionnaire (HUI)	2
PROMlS	1
Likert Scale	1
EORTC-QIL-30	1
Ulm QoL Inventory for Parents	1
Caregivers Burden Inventory	1
McGill QoL questionnaire (revised)	1
WH0-5	1
PKU-QoL	1
INCAT Disability Scale	1
Krepp's Fatigue Severity Scale	1
Beck Depression Inventory	1
KIDS' ITP Tools	1
Hunter Syndrome Functional Outcomes for	1
Clinical Understanding Scale	
Childhood Health Assessment	1
Questionnaire (CHAQ)	—
Child Health Questionnaire Parent Form 50	1
Zung 's Self-Rating Anxiety Scale	1
Zung's Self-Rating Depression Scale	1
Hert Hope Index	1
Kidscreen -27	1
Multi Dimensional Scale of Perceived	1
Social Support	—

## Discussion

The literature reviewed in the scope of this paper shows that, when used to assess the impact of rare diseases, COI studies are generally based on a societal perspective, which health economists identify as the most complete approach (Jo, 2014; Café et al., 2019). A minority of reviewed COI studies (11%) used a patient/family perspective. However, although a patient’s perspective can underestimate the impacts of rare diseases for society, they can represent an interesting highlight of the impacts, both social and economic, borne by people who are directly affected by the diseases in question, including on diseases for which there is no treatment and/or for which informal care needs are especially high, while this becomes relevant from a societal perspective when considered collectively. For instance, Galvin et al. (Galvin et al., 2020) report that people living with inherited retinal diseases (IRDs) in the Republic of Ireland (ROI) and the United Kingdom (UK) incur the largest share of the costs related to these diseases (51% in ROI and 36% in UK), while society incurs 17 and 9% of the costs related to IRDs. The EveryLife Foundation’s study (EveryLife Foundation, 2021) corroborates these findings, although this study focused mostly on the important economic impact borne by the US health system. As patients are often the ones bearing most of the financial, health and psychological burden (Navarrete-Opazo et al., 2021), more COI studies using a patient/family perspective could help better define the impact of rare disease from the individual point of view, although being more difficult and time consuming to conduct than from a societal perspective. Going further, the vast majority of COI studies use a prevalence-based estimation, meaning that costs were retrieved and analysed for typically a 1-year period. A large part of rare diseases being chronic, it could be argued that these analyses - valid at a certain point in time - may not reflect accuracy at a later stage of a defined disease. The progressive and changing nature of certain conditions, such as Huntington disease, also adds challenging elements to the analysis, including an accurate staging of the disease at a specific point in time, the time-related effects of its treatment or the aspects of continuity of care that could overall improve the HRQoL of people living with rare long-term disorders (Calvert et al., 2013; van Walsem et al., 2017). Similar conclusions could be drawn with regards to the inadequacy of COI studies to take account of the benefits of treatments (Linertová et al., 2017). As for the content of COI studies, several observations can be made. The National Economic Burden of Rare Disease Study (EveryLife Foundation, 2021) in the United States is so far the first attempt to investigate the burden of a large group of diseases (379), encompassing direct medical, indirect and non-medical costs. As mentioned earlier, the BURQoL-RD project in Europe used a similar approach regarding the costs taken into consideration but was limited to 10 rare diseases (López-Bastida et al., 2016a), while García-Pérez et al. (García-Pérez et al., 2021) report some diversity in the costs included in the studies they reviewed, all of them including direct medical costs in their analysis but only 60, 69 and 43% including respectively non-medical costs, loss of productivity and costs of informal care. As Angelis et al. (Angelis et al., 2015) and Armeni et al. (Armeni et al., 2021) point, there exists significant heterogeneity in the identification of costs associated with rare diseases, as well as in the methodologies used to do so. This in turn complicates comparison between studies. Moreover, there seems to be a tendency in COI studies to leave certain side effects of diseases (e.g. physiotherapeutic care, psychological follow up) out of the scope of direct medical costs while focussing mostly of diagnostic tests, hospitalisation and medical treatment, thus quite overlooking the multilevel impact of rare disease and types of care beyond clinical options. Nevertheless, there seems to be general agreement that, taken together, indirect, and non-medical costs tend to exceed direct medical costs (EveryLife Foundation, 2021), although current studies are not large enough in terms of scope, either geographically or disease wise, to be truly reflective of the complexity of all rare diseases and their impacts. Lastly, the review of the current literature shows that some diseases - such as haemophilia, Duchenne muscular dystrophy (DMD) or cystic fibrosis for instance - are more salient in the literature than others (García-Pérez et al., 2021). In Europe, this could be explained by the selection of 10 diseases included in the BURQoL-RD project (López-Bastida et al., 2016a), which used a Delphi approach combined with a Caroll Diagram to select a representative set of rare diseases based on three criteria: prevalence, availability of effective treatment and need for care. The availability of COI studies being positively correlated to the existence of a treatment (Armeni et al., 2021) and knowing that 95% of rare diseases currently do not have a treatment available (Sequeira et al., 2021), it could be argued that a vast majority of conditions are thus left outside the scope of COI and that there is a need to go beyond the existence of a treatment to conduct COI studies to illustrate the true impact of rare diseases. The paucity of available treatments further emphasises the importance of adopting a holistic approach when investigating the impact of rare diseases to avoid leaving out of scope conditions for which there are no medical options but for which daily impacts remain high. Similarly there is a necessity to consider investigating other diseases than the ones that are most salient in the literature. The appropriateness of COI methodologies and the use of its outputs in regulatory decisions could also be questioned in several cases. For instance, a treatment for Sickle Cell Disease that has been administered to a small number of patients in the US and has so far shown outstanding results as patients enrolled in the trial have been declared free of the disease, is expected to reach a price tag of $1 to $2 million per patient for a single dose (Kolata, 2014). While undoubtedly representing a high economic burden, it is however argued that the price of a single dose could be less than the price paid for the lifetime management of the disease, which may not be reflected in COI analyses nor in regulatory decisions based on economic evaluation of treatments for rare diseases.

On the other hand, intangible costs are typically measured in loss of quality of life (Xie et al., 2008). Rare diseases are known to have a high impact, beyond medical symptoms, on quality of life (Uhlenbusch et al., 2019a) and their impact on pain, mental health and overall wellbeing is often important, as reported by EURORDIS in their Juggling Care Survey (Eurordis, 2017). This is further exacerbated by the often-chronic characteristics of these diseases and social, economic, and psychological impacts inherent to rare diseases (Alonso et al., 2004). With regards to mental health for instance, anxiety and depression are often reported amongst people living with a rare disease in addition to condition-specific symptoms (Winter et al., 2010; Uhlenbusch et al., 2019b; Uhlenbusch et al., 2021). Similar conclusions can be drawn for diagnosis, as people living with a rare disease often wait on average 5 years before receiving a correct diagnosis, thus contributing to the psychological impact of rare diseases in addition to costs incurred during the diagnostic odyssey (European Brain Council, 2017; Eurordis, 2017; World Economic Forum, 2020). However, it appears that intangible costs are less consistently included in the study of the impact of rare diseases than direct medical costs or loss of productivity. The rare attempts, such as the BURQoL-RD project, that included both COI and HRQoL seem to have put the main emphasis on tangible costs and the recent study by EveryLife Foundation solely focussed on direct medical, indirect, and non medical costs, leaving aside an important aspect of rare diseases and therefore linking the impact of rare diseases to economy alone. In addition, this review highlights that, while many instruments could be used to evaluate HRQoL, a vast majority of these instruments are generic. The EQ-5D questionnaire, for instance, is said to be one of the most prevalent ways to measure HRQoL worldwide (Zhou et al., 2021). However, it could be argued that these generic measurement tools are limited to a certain number of health dimensions and may therefore miss important aspects of QoL to the profit of general health status regardless of disease’s specificities (Mossman et al., 2016; Efthymiadou et al., 2019). Despite the salience of some tools adapted for defined disease areas (e.g. PedsQL and OHIP-14) it could thus be argued that the use of disease-specific measurements and condition-specific questionnaires may be more appropriate when assessing QoL amongst people living with rare diseases than generic questionnaires, mostly due to the complexity, often chronic character and low prevalence of these conditions. Initiatives, such as the IMPACT survey conducted by Osteogenesis Imperfecta Federation Europe (Osteogenesis Imperfecta Federation Europe, 2022), show that there exist attempts to design measurement tools more adapted to specific pathologies and that the expertise and experience of people living with these diseases could greatly contribute to the creation of these tools. Matching COI estimations with QoL analyses could contribute to more appropriate and accurate procedures in HTAs or clinical trials for rare disease. However, our findings showed that few attempts have been made to match these measures of disease burden or analyse the relationships between formal and informal healthcare resources and QoL. Kodra et al. (Kodra et al., 2014) demonstrated that better QoL among people living with haemophilia in Italy had a positive impact on consumed healthcare and non-healthcare resources, with a reduction in costs. Although their study focuses on cancer, the findings of Mausbach et al. (Mausbach et al., 2020) may explain the relationship between direct medical costs and QoL by linking anxiety and depression among patients to higher risks of hospitalisation, longer hospital stays, and overall accrued higher healthcare costs. Liu et al. (Liu et al., 2018) also established a link between poorer QoL and higher economic impacts among people living with acromegaly, while Bogart and Irvin (Bogart and Irvin, 2017) hold that early diagnosis is also a component of improved HRQoL among people with rare disorders. In addition, for diseases for which there is currently no treatment, informal care and non-healthcare costs represent the major drivers for QoL, further complicating the analysis of the relationships between QoL and healthcare and non-healthcare costs (Kodra et al., 2020). Hand in hand, a holistic approach matching COI and HRQoL features is needed to draw a comprehensive picture of all aspects and challenges faced by people living with these rare conditions. As the findings show, there exist studies that survey both aspects, but the analysis of the potential relationships between them is currently missing, despite authors reporting these knowledge gaps and their consequences for people living with rare disease in accessing the level of health and social care that they need (Winter et al., 2010; Calvert et al., 2013; van Walsem et al., 2017). In addition, the complexity and heterogeneity of rare diseases further complicates research processes, mostly as their chronic character is likely to greatly impact the continuous and varying relationship between QoL and costs. Although focussing solely on HRQoL for haemophilia A, Poon et al. (Poon et al., 2014) advocate the use of multivariable multilevel (MVML) modelling to account for time-invariant and time-varying factors affecting HRQoL, and thus the chronic nature of rare diseases. However, they did not explore how this model could be further coupled with economic analysis, and no other article retrieved in this scoping review addressed MVML modelling nor other economic analysis such as cost-utility analysis (CUA), which compares costs with outcomes expressed in quality-adjusted life years, a measure combining both length and quality of life (Whitehead and Ali, 2010).

## Conclusion

As Armeni et al. (Armeni et al., 2021) state, COI studies in rare diseases are still scarce in comparison to economic evaluations regardless of the perspective used. Despite increasing attention being paid to rare diseases and their impacts on society, healthcare systems and individuals, a review of the literature shows a main emphasis placed on economic features, with COI studies being the reference to define the burden of disease and influence political decisions. However, the impact of rare diseases go well beyond economic terms and their impacts significantly affects the quality of life of people living with a rare disease and their carers, which tends to and should be measured beyond the loss of productivity—for example level of pain, discomfort or mental health. Moreover, the burden of (rare) disease remains siloed between disciplines and therefore does not provide us with a comprehensive picture of the impact of disease nor the impact for all rare diseases, while the lack of reliable and sufficient data further complicates research on this topic. Hand in hand, several observations can be made. Firstly, Europe currently lacks a comprehensive review of impact assessment across rare diseases and countries that would help understanding the extent to which rare conditions affect individuals, society and healthcare systems. Initiatives such as the National Economic Burden of Rare Disease Study (EveryLife Foundation, 2021) or the study commissioned by Chiesi Farmaceutici (Andreu et al., 2022), applied to the European context would not only contribute to a better understanding of the various and often considerable impacts rare disease represent to the rare disease community and society, but would also provide all relevant stakeholders with a solid knowledge base to justify further investments in research and development, accessibility to treatments and disease management in line with Europe’s ambition to remain a leader in innovation and in the respect of basic human rights. Secondly, methods used to assess the impact of rare diseases have been shown to differ greatly from one research discipline to another and to be conducted in siloes. Rare diseases having a multitude of different and severe impacts, research on this topic must be better adapted to more adequately cover the full scope of what impacts mean to all relevant stakeholders, from economic considerations to physical, psychological and social impacts primarily endured by people living with a rare disease and their families/carers, thus accounting for QoL and economic costs equally. Consensus on methodology is therefore needed to allow for comparison of results and must include non-economic evaluations to effectively provide decision-makers with robust data and information in a context of rising healthcare competing priorities and limited resources to be allocated. Thirdly, with a vast majority of rare diseases having no treatment available, assessment methods of the impact of rare conditions must go beyond the existence of a pharmaceutical option and include the assessment of alternative ways to care, interventions and disease management. In doing so, and ideally including people living with a rare disease in the assessment of care and health technologies, future HTA would be better adapted to respond to the needs of the rare disease community and its stakeholders, with the implementation of value-based criteria defined by a comprehensive assessment of diseases’ impact and management going beyond economic terms and incorporating important aspects that are currently not taken into account in decision making processes based on HTA outcomes.

Hand in hand and based on the findings of this scoping review, authors of this paper therefore conclude that a comprehensive study on impact of rare disease across countries in Europe is lacking and is needed to better plan and attract private and public investments in the research and development of rare disease therapies—for which there is a significant unmet need and a great potential for impact. They also state that existing studies are heterogeneous in their scope and methodology and often lack a holistic picture of the impact of rare disease, as they focus on the economic impact and ignore the social and psychological. A consensus on standards and methodology across countries and diseases will thus be helpful in better applying such an evidence base. Lastly, studies that do consider a holistic approach are often conducted by pharmaceutical companies and patient organisations exploring a specific disease area but not necessarily visible in the literature and could benefit from the sharing of standards and best practices to better achieve their impact assessments and apply them in each stage of the therapeutic life cycle.
